# Unveiling the intermediate tumour: a case report of uterine smooth muscle tumour of uncertain malignant potential and review of magnetic resonance imaging features

**DOI:** 10.1093/bjrcr/uaaf053

**Published:** 2025-11-09

**Authors:** Phoebe Hay Pui Yeung, Siu Chun Wong, Ting Wong, Yuen Fun Mak, Ka Yu Cheng, Yat Yee Shizuka Chan, Tsz Man Mimi Fung

**Affiliations:** Department of Diagnostic and Interventional Radiology, Princess Margaret Hospital, Kowloon West Cluster, Kowloon, Hong Kong SAR; Department of Diagnostic and Interventional Radiology, Princess Margaret Hospital, Kowloon West Cluster, Kowloon, Hong Kong SAR; Department of Diagnostic and Interventional Radiology, Princess Margaret Hospital, Kowloon West Cluster, Kowloon, Hong Kong SAR; Department of Pathology, Princess Margaret Hospital, Kowloon West Cluster, Kowloon , Hong Kong SAR; Department of Obstetrics and Gynaecology, Princess Margaret Hospital, Kowloon West Cluster, Kowloon , Hong Kong SAR; Department of Obstetrics and Gynaecology, Princess Margaret Hospital, Kowloon West Cluster, Kowloon , Hong Kong SAR; Department of Obstetrics and Gynaecology, Princess Margaret Hospital, Kowloon West Cluster, Kowloon , Hong Kong SAR

**Keywords:** uterine smooth muscle tumour of uncertain malignant potential, magnetic resonance imaging, gynaecological imaging

## Abstract

Uterine smooth muscle tumour of uncertain malignant potential (STUMP) is a rare and diagnostically challenging entity, lying on the spectrum between benign leiomyomas and malignant leiomyosarcomas. Its unpredictable biological behaviour and potential for metastasis and progression to leiomyosarcoma necessitate surgical removal and long-term surveillance for late recurrence and malignant transformation. We present a case of uterine STUMP in a 49-year-old woman with a large uterine mass exhibiting suspicious magnetic resonance imaging (MRI) features preoperatively. She underwent a hysterectomy with histological confirmation of STUMP. Through radiologic-pathologic correlation, we highlight key MRI features that can suggest the diagnosis of STUMP and aid in differentiating it from benign leiomyomas. While differentiating STUMP from leiomyosarcoma on imaging remains challenging due to its rarity and overlapping imaging characteristics, imaging plays a crucial role in detecting complications and guiding follow-up. Multidisciplinary management in tertiary centres with gynaecologic oncology expertise is essential for comprehensive patient care.

## Case presentation

A 49-year-old, multiparous woman with an unremarkable past medical history presented with progressive lower abdominal distension of 18 months’ duration. She reported regular menstrual cycles without dysmenorrhea. Physical examination revealed a large pelvic mass extending to the level of the umbilicus. Transabdominal ultrasound demonstrated a heterogeneous uterine mass of at least 12 cm in size. MRI was subsequently performed for further characterisation.

## Imaging findings

MRI of the pelvis was performed on a 1.5 T scanner (Siemens Magnetom Sola 1.5 T MRI scanner). The protocol included T2-weighted images in three orthogonal planes, diffusion-weighted imaging (DWI) with *b*-values of 50 and 1200 s/mm^2^ and corresponding apparent diffusion coefficient (ADC) map, and T1-weighted images before and after intravenous gadolinium contrast administration in the axial plane.

**Figure 1.**
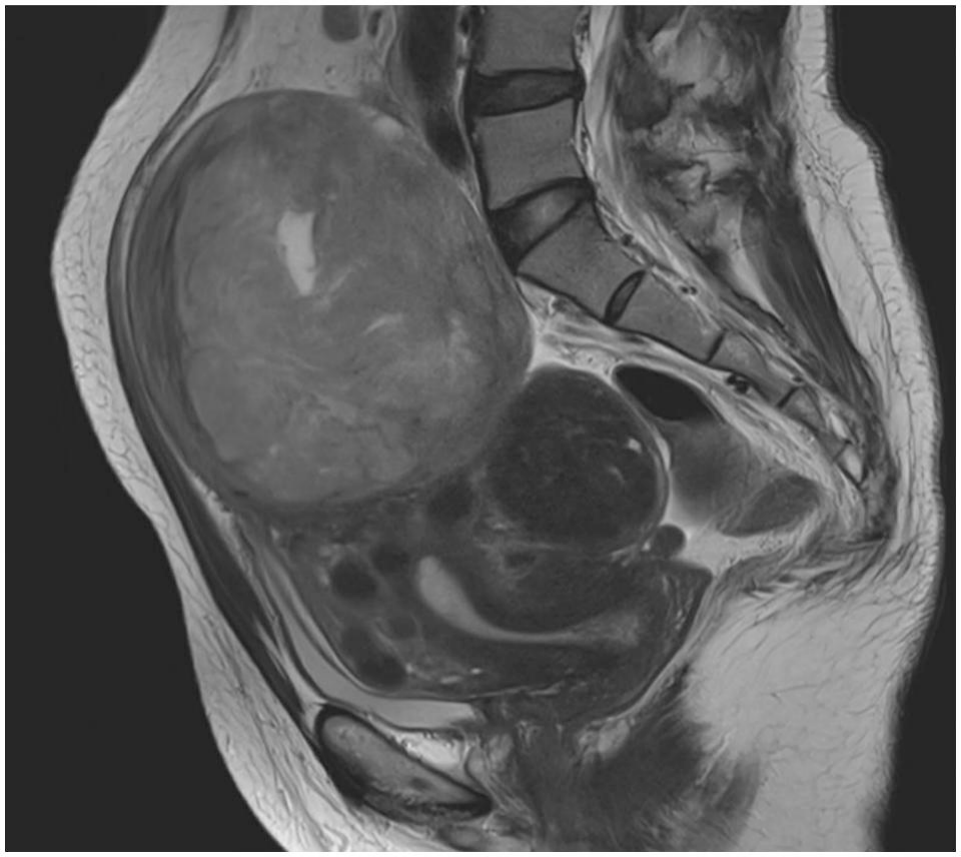
Sagittal T2W image depicts a large heterogeneously hyperintense mass at uterine fundus.

**Figure 2.**
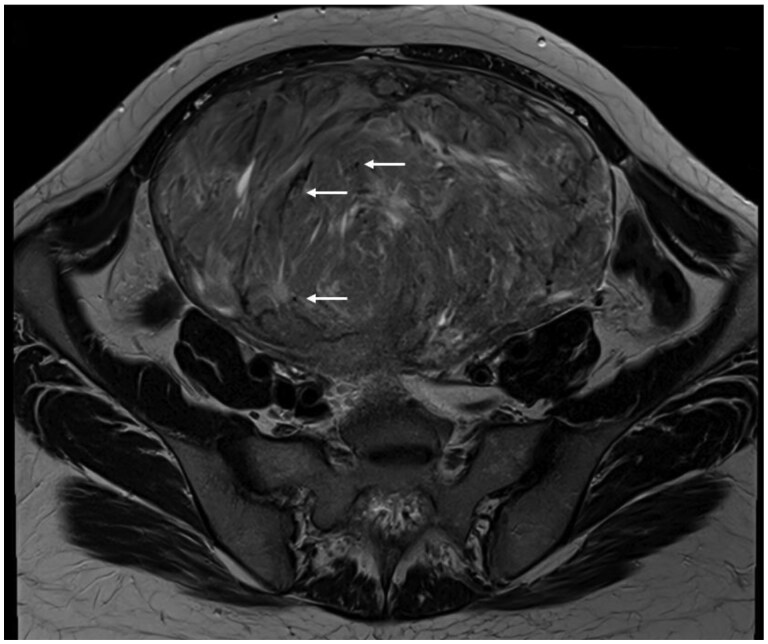
Axial T2W images demonstrate tortuous flow-related signal voids (narrow arrows) seen within the lesion.

**Figure 3.**
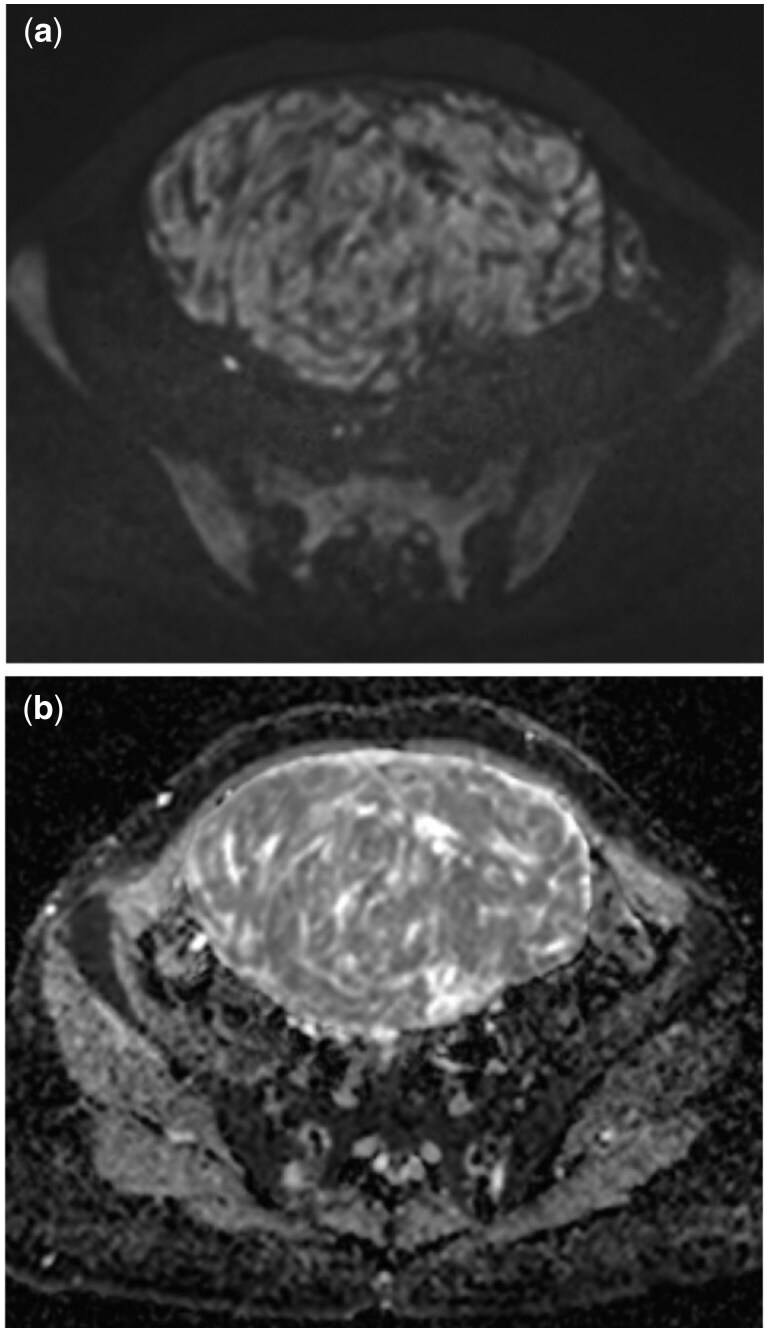
(A) High b value (1200 s/mm^2^) DWI image demonstrates hyperintense signal on DWI similar to that of endometrium (endometrium not included on current image). (B) ADC image demonstrates corresponding hypointense signal suggesting restricted diffusion.

**Figure 4.**
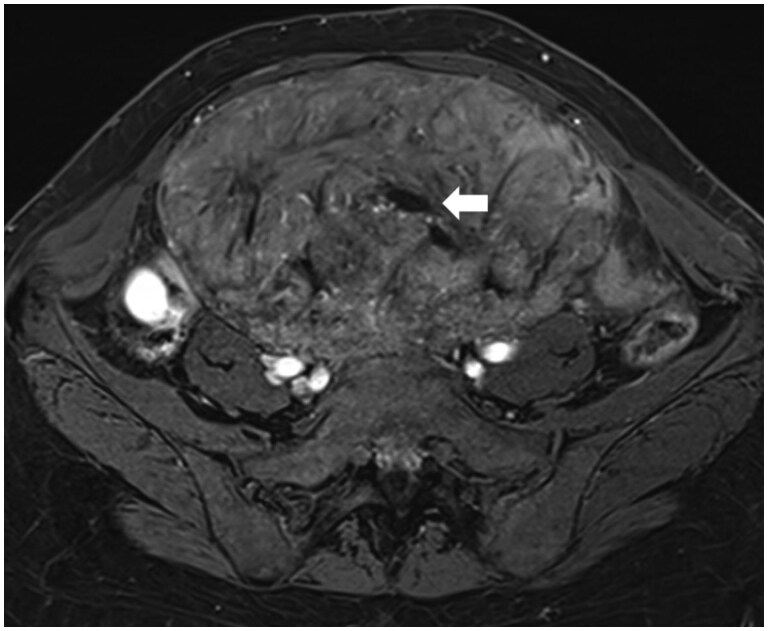
Post contrast T1W image with subtraction reveal central non enhancing areas (bold arrow), which can be tumoural necrosis.

MRI revealed a markedly enlarged uterus containing multiple myometrial masses. The dominant mass, located in the uterine fundus, measured 15 × 10.6 × 11.7 cm in size. This mass demonstrated hypointensity on T1-weighted (T1W) images and heterogeneous hyperintensity on T2-weighted (T2W) images (Figure 1). Prominent tortuous flow voids were observed within the lesion on T2W images, suggestive of significant vascularity (Figure 2). Post-contrast imaging revealed heterogeneous hyperenhancement relative to the surrounding myometrium, with central non-enhancing areas which can be tumoural necrosis (Figure 4). Areas of restricted diffusion were identified, with a hyperintense signal on high b-value DWI images similar to that of the endometrium, indicating increased cellularity (Figure 3). No extraserosal tumour extension. These findings raised suspicion for a myometrial mass with hypervascularity and increased cellularity, with the primary differential diagnoses being smooth muscle tumour of uncertain malignant potential (STUMP) and uterine leiomyosarcoma.

Additional findings included multiple smaller typical uterine leiomyomas. A 4 cm right ovarian endometrioma and a right peritoneal inclusion cyst adjacent to the fallopian tube were also present. The left ovary was normal. No suspicious pelvic or retroperitoneal lymphadenopathy was identified.

## Treatment and outcome

Given the concerning imaging characteristics of the dominant myometrial mass, the patient was counselled regarding the possibility of malignancy and the recommendation for total hysterectomy. She elected to proceed with total abdominal hysterectomy with bilateral salpingo-oophorectomy (TAHBSO). Intraoperative findings included an enlarged uterus consistent with an 18-week gestational size. A 15 cm pedunculated myometrial mass was identified arising from the fundus and exhibiting adhesions to the omentum in the left fundal region. Several smaller fibroids (up to 4 cm) were also noted. The right ovary contained a 4 cm endometrioma with an adjacent peritoneal inclusion cyst, consistent with preoperative imaging.

Final histopathological analysis confirmed a diagnosis of STUMP. Microscopic examination revealed a hypercellular tumour composed of spindle cells, without significant nuclear atypia nor rare mitotic figures. Coagulative necrosis was present, and some vessels within the fundal tumour exhibited fibrinoid necrosis and thrombotic changes.

The patient had a smooth postoperative recovery and remained asymptomatic at a follow-up 2 months after the operation. Her baseline follow-up CT of the thorax, abdomen, and pelvis at 4 months postoperatively showed no residual or recurrent pelvic tumour.

## Differential diagnosis and discussion

STUMP represents an intermediate group of heterogeneous uterine smooth muscle tumours, exhibiting atypical histological features that do not meet the criteria for either benign leiomyoma or leiomyosarcoma according to World Health Organization (WHO) criteria.^1^ STUMP is an under-recognised entity compared with its benign and malignant counterparts, accounting for approximately 0.01% of all surgically treated uterine smooth muscle tumours.^2^

STUMP is more commonly observed in women in their forties^3^^,^^4^ with clinical manifestations similar to other uterine masses, including menorrhagia and pelvic mass.^3^ These tumours exhibit unpredictable biological behaviour, ranging from indolent to potentially aggressive.^5^ Potential exists for distant metastasis^5^ and transformation to or recurrence as leiomyosarcoma.^3^^,^^6^ The reported recurrence rate for STUMP ranges from 10% to 27%, with known potential for late recurrences beyond 5 years.^7^ Therefore, the diagnosis of STUMP mandates further imaging investigations to exclude distant metastasis and long-term imaging surveillance to detect late recurrence.

Histological assessment is the cornerstone of STUMP diagnosis, requiring exclusion of benign leiomyoma and leiomyosarcoma^8^ based on the degree of mitotic count, cytological atypia, and evidence of necrosis. The Stanford criteria^9^ for diagnosing leiomyosarcoma require the presence of at least 2 out of 3 indicators: marked nuclear atypia, high mitotic activity ≥10 mitoses/10 high power field (HPF), and coagulative tumour cell necrosis. Benign leiomyoma is diagnosed in the absence of necrosis or atypia and with <4 mitoses/10 HPFs. Tumours falling between these two categories, previously labelled as smooth muscle tumours of low malignant potential, demonstrate a low mitotic index (<10/10 HPF), up to mild atypia, and the presence of coagulative tumour cell necrosis.^4^ The WHO classification categorises smooth muscle tumours exhibiting potentially malignant features without fulfilling all criteria for leiomyosarcoma or benign leiomyoma as STUMP. Studies have explored the diagnostic value of immunohistochemical markers (eg, p16, p53, Ki-67) and molecular testing (eg, *MED12* mutations, FH deficiency) in differentiating STUMP from other tumours; however, their current role remains ancillary due to overlapping expression of these markers between benign and malignant uterine smooth muscle tumours.^6^

Preoperative imaging diagnosis of STUMP is challenging due to the rarity of the condition, non-specific appearance, and the tendency to report STUMP characteristics within the broader category of malignant leiomyosarcoma. On ultrasonography, STUMP has a variable appearance, ranging from hypoechoic to hyperechoic with a heterogeneous echotexture.^3^^,^^8^

Pelvic MRI is the preferred modality for characterising indeterminate uterine masses due to its superior soft tissue contrast, multiparametric assessment with enhancement and diffusion characteristics, multiplanar image acquisition, large field of view, and lack of ionising radiation.^10^ Current understanding of MRI features of STUMP is primarily based on case series with a limited number of cases.^10^

Reported MRI findings of STUMP include heterogeneous contents with variable sizes that may exceed 10 cm in diameter. The presence of mixed solid and cystic components with necrotic areas and irregular septa has also been described. Hyperintense signal on T2WI, involving more than half of the tumour volume, helps distinguish STUMP from typical leiomyomas, which demonstrate a predominantly hypointense signal on T2WI. Intralesional hyperintense T1W signal areas can indicate haemorrhage or necrosis. Restricted diffusion and heterogeneous enhancement have also been reported in STUMP tumours.^11^

According to the latest expert consensus by Hindman et al. in 2022, a proposed diagnostic algorithm for evaluating uterine myometrial masses suggests that if a mass exhibits hyperintense T2W signal with contrast enhancement, further characterisation with lesion signal intensity at high b-value images (*b*-value ≥1000 s/mm^2^) of DWI can be helpful. Lesions with signal intensity equal to or higher than that of the endometrium and ADC values below 0.9 × 10^−3^ mm^2^/sec should raise suspicion for STUMP or leiomyosarcoma.^10^ It is also important to consider that absolute ADC cut-off values can vary depending on the MRI system vendor and specific imaging parameters used, which may affect reproducibility and interpretation.^12^ Thus, these findings should be integrated with other clinical and imaging features to make a comprehensive diagnostic judgment.

Frank extra uterine tumour invasion, suspicious lymphadenopathy or metastatic deposit would be supportive features of malignant leiomyosarcoma.

Uterine leiomyosarcoma is a rare but highly aggressive malignant tumour that arises from the myometrium. It typically presents in postmenopausal women and accounts for a significant proportion of uterine sarcomas. Clinically, patients may experience a rapidly enlarging uterine mass, pelvic pain or abnormal uterine bleeding.^13^ Imaging features often include a large, heterogeneous lesion with areas of necrosis, irregular margins and increased vascularity. On T1W images, lesions appear heterogeneous and hypointense, reflecting haemorrhage and necrosis. T2W images reveal intermediate to high signal intensity with central hyperintense necrotic areas.^13^ Their margins tend to be irregular and ill-defined, indicating infiltrative growth. On DWI, leiomyosarcomas display strong diffusion restriction with low ADC values due to high cellularity. Contrast-enhanced MRI usually shows early heterogeneous enhancement caused by necrosis and haemorrhage.^13^ These tumours are often larger and display rapid growth compared to leiomyomas. However, overlap with degenerating leiomyomas can occur; features such as irregular margins, necrosis, and diffusion restriction should raise suspicion toward malignancy.^14^ Histologically, they are characterised by marked nuclear atypia, high mitotic activity, and tumour necrosis, distinguishing them from benign leiomyomas. Due to their aggressive nature, leiomyosarcomas have a poor prognosis, with a high likelihood of local recurrence and distant metastases, particularly to the lungs and liver.^15^

On the other hand, benign leiomyoma, the most common benign uterine tumours, manifests as well-circumscribed masses with hypointense signals on T2W images due to their dense smooth muscle content. On T1W images, they exhibit hypo to isointense signals, without haemorrhage unless degenerative changes occur. Leiomyoma variants such as cellular or necrotic leiomyomas may have hyperintense T2W signal areas. These generally do not demonstrate restricted diffusion on DWI. Post contrast tumoural hypoenhancement is typical, but variable enhancement may be seen in degenerated leiomyomas.^16^ These features help distinguish benign leiomyomas from more aggressive lesions.

Other rarer differential diagnoses include rare uterine sarcoma such as stromal sarcoma. Differentiation and diagnosis of these rare entities ultimately requires pathological confirmation.

In our case, the large 15 cm tumour exhibited hyperintense T2W signal with moderate restricted diffusion with similar DWI signal to that of the endometrium on high b-value images, consistent with previously suggested MRI features of STUMP. This prospectively validates the usefulness of the diagnostic algorithm proposed by Hindman et al in 2022. The tumour hypercellularity accounts for the moderate restricted diffusion on MRI.

The index tumour demonstrated a plethora of flow-related signal voids from underlying vasculature, indicating tumour hypervascularity. This specific feature has not been previously reported as a distinct MRI characteristic of STUMP, to the best of our knowledge. This finding may corroborate previously reported sonographic findings of hypervascularity and Doppler enhancement in malignant uterine soft tissue tumours.^5^ Central non-enhancing areas on MRI correlated with histological findings of necrosis.

Histological findings of coagulative necrosis without significant mitosis or atypia established the diagnosis of STUMP.

Multidisciplinary team collaboration involving gynaecologic oncologists, pathologists, and radiologists is essential for diagnosis, treatment planning considering the patient’s age and fertility desires, identification of high-risk histological features (eg, coagulative tumour cell necrosis^6^ and significant atypia), and designing tailored follow-up surveillance plan.

No international guidelines regarding the management of STUMP have been published to date due to the rarity of this condition. Current management primarily involves surgical resection. Myomectomy may be considered in patients desiring future fertility, while hysterectomy is indicated when fertility is no longer desired or as completion surgery to reduce recurrence risk, particularly in those with positive margins or concerning histologic features after prior myomectomy.^5^

MRI is helpful in assessing local recurrence in the uterus after myomectomy, while cross-sectional imaging with annual CT scans of the thorax, abdomen, and pelvis have been suggested to detect any distant metastasis.^5^

## Conclusion

In summary, STUMP has garnered increasing attention as an intermediate category between benign leiomyoma and malignant leiomyosarcoma, exhibiting unpredictable biological behaviour with potential for metastasis, late recurrence, and degeneration into leiomyosarcoma. MRI can differentiate STUMP from benign tumours based on T2 signal heterogeneity, restricted diffusion, necrotic areas, and ADC value analysis. However, differentiation of STUMP from leiomyosarcoma remains challenging in the absence of extrauterine tumour spread, due to the rarity of STUMP and the frequent grouping of its features under the broader category of malignancy in the literature. We contribute new insight to the current literature by demonstrating tumour hypervascularity with a plethora of flow-related signal voids and avid contrast enhancement in this case, a feature not previously reported on MRI for STUMP. Management is primarily based on multidisciplinary decisions and surgical resection, considering the patient’s age and fertility desires.

## Learning points, take home message

- Uterine smooth muscle tumours present a diagnostic spectrum from benign leiomyomas to malignant leiomyosarcomas, with STUMP representing a challenging intermediate category requiring careful radiological pathological correlation.
- While STUMP is rare, it may be underrecognised due to overlapping imaging features with cellular leiomyomas and early-stage leiomyosarcomas; MRI plays a pivotal role in preoperative characterisation.
- STUMP carries a risk of recurrence and progression, necessitating complete surgical excision with thorough histopathological evaluation and long-term imaging surveillance even after resection due to potential late recurrences.
- Key MRI features raising suspicion of STUMP include heterogeneous T2 signal, diffusion restriction, presence of necrotic areas and hypervascularity.
